# Deciphering mechanisms of immune escape to inform immunotherapeutic strategies in multiple myeloma

**DOI:** 10.1186/s13045-022-01234-2

**Published:** 2022-02-16

**Authors:** Muthulekha Swamydas, Elena V. Murphy, James J. Ignatz-Hoover, Ehsan Malek, James J. Driscoll

**Affiliations:** 1 Seidman Cancer Center, University Hospitals, Cleveland, OH USA; 2 Department of Biochemistry, Case Western Reserve University, Cleveland, OH USA; 3 Case Comprehensive Cancer Center, Hematopoietic and Immune Cancer Biology Program, Cleveland, OH USA

**Keywords:** Multiple myeloma, Immunotherapy, Immune escape, Drug resistance, Proteasome, Cytotoxic T cell, NK cell, CAR T cells

## Abstract

Multiple myeloma is an incurable cancer characterized by the uncontrolled growth of malignant plasma cells nurtured within a permissive bone marrow microenvironment. While patients mount numerous adaptive immune responses directed against their disease, emerging data demonstrate that tumor intrinsic and extrinsic mechanisms allow myeloma cells to subvert host immunosurveillance and resist current therapeutic strategies. Myeloma downregulates antigens recognized by cellular immunity and modulates the bone marrow microenvironment to promote uncontrolled tumor proliferation, apoptotic resistance, and further hamper anti-tumor immunity. Additional resistance often develops after an initial clinical response to small molecules, immune-targeting antibodies, immune checkpoint blockade or cellular immunotherapy. Profound quantitative and qualitative dysfunction of numerous immune effector cell types that confer anti-myeloma immunity further supports myelomagenesis, disease progression and the emergence of drug resistance. Identification of tumor intrinsic and extrinsic resistance mechanisms may direct the design of rationally-designed drug combinations that prevent or overcome drug resistance to improve patient survival. Here, we summarize various mechanisms of immune escape as a means to inform novel strategies that may restore and improve host anti-myeloma immunity.

## Background

Multiple myeloma (MM) is a malignant neoplasm characterized by the progressive growth and proliferation of clonally-transformed plasma cells (PCs) that reside within bone marrow (BM) [1–3]. MM is the second most common hematologic malignancy with > 160,000 new cases occurring every year globally and ~ 35,000 per year in the U.S [4, 5]. MM follows a multistep process that includes tumor immune escape and the accumulation of genomic alterations within the malignant clone(s) that drive progression from precursor stages, i.e., monoclonal gammopathy of unknown significance (MGUS) and smoldering MM (SMM) [4, 6–8]. Overt MM is characterized by the accumulation of clonal PCs within BM that promotes end organ dysfunction clinically recognized as anemia, lytic bone disease, hypercalcemia, and renal injury [1, 7].

Modern myeloma therapy has greatly improved patient outcomes with 5-year overall survival (OS) nearly doubling from 32% in 1996 to 54% in 2020 [9]. In addition to immunomodulatory drugs (IMiDs) and proteasome inhibitors (PIs), the anti-CD38 antibody (Ab) daratumumab was approved for relapsed and/or refractory MM (RRMM) and has moved into the frontline setting for newly diagnosed MM (NDMM) [10–14]. Despite these improvements, MM remains incurable, and further research into myelomagenesis and drug resistance is needed [10–12]. Of particular interest is the tumor microenvironment (TME) and mechanisms of immunological escape that foster disease progression [13, 14]. Here, we discuss tumor intrinsic and extrinsic mechanisms of immune escape that promote resistance to anti-myeloma immunity.

## Tumor-intrinsic mechanisms of immune escape in myeloma

Recent pre-clinical and clinical studies support the strategy that triggering host immunity is critical for most anti-myeloma therapies to be effective [11–14]. PIs, IMiDs, monoclonal Abs (mAbs), autologous stem cell transplantation (ASCT), T cell-based immunotherapy, e.g., chimeric antigen receptor (CAR) T cells and bispecific T cell engagers (BiTEs), have shown clinical benefit in RRMM [15, 16]. MM is characterized by disrupted immune surveillance, impaired Ab production, deregulated T and NK cell compartments, disrupted antigen presentation, upregulation of inhibitory surface ligands, and recruitment of immunosuppressive cells [17].

### Immunoediting

Immunosurveillance specifies the host immune reaction against tumor cells while immune escape refers to tumor cell evasion of host immunity [18, 19]. Immune escape in MM is driven by immunoediting in which the immune system protects the host [20–22]. However, the immune system also places evolutionary pressure on malignant cells causing them to undergo immunogenic sculpting that enables immune escape and disease progression [23, 24].

Immunoediting proceeds through the three phases; elimination, equilibrium, and escape. During elimination, transformed cells that have escaped normal cell-intrinsic apoptotic/senescence checkpoints are recognized and killed by cells of the innate and adaptive immune systems. In the equilibrium stage, tumor subclones that survived elimination through the acquisition of additional genetic alterations, begin to expand. However, overall net tumor growth is prevented primarily by adaptive immunity [23, 25]. Over time, the evolutionary pressure placed on the developing tumor promotes the selection and expansion of tumor subclones. In the last stage, tumor outgrowth is no longer restricted by host immunity and tumor subclones emerge evidenced by clinically apparent disease. While the immune system is capable of recognizing and killing MM cells to constrain tumor growth, the same mechanism also promotes the emergence of malignant subclones [26].

### Loss of antigenicity

Immunosurveillance is dependent on the recognition of tumor antigens and loss of tumor antigenicity is observed in MM [27]. Lack or loss of tumor antigenicity represents a key mechanism of immune escape and resistance to T cell-based immunotherapies. During myelomagenesis, aberrant expression of cancer-related genes and protein products initially promotes a cellular immune response. Reduced or defective expression of tumor antigens or major histocompatibility complexes (MHC) along with defects in antigen processing and presentation help tumor cells escape cytotoxic T lymphocytes (CTLs) [28, 29]. Elucidation of immune deficits that promote cancer progression has been difficult and lacks a unifying mechanism [28, 30, 31].

Loss of antigenicity through MHC class I downregulation or changes in tumor-associated antigen epitopes affect CTL responses in cell lines. Studies using murine models and clinical trials demonstrate that this phenomenon impacts anti-cancer treatment strategies [27, 29, 31]. High, uniform expression of CD38 by myeloma cells, combined with its role in cell signaling, demonstrates that CD38 is a viable therapeutic target in myeloma patients. Ise et al. reported loss of CD38 on MM cells in RRMM patients [32]. Loss-of-function of γ-aminobutyric acid receptor-associated protein (GABARAP) has also been identified as a tumor-intrinsic mechanism of resistance to bortezomib-induced cell death [33]. Lozano et al. reported that levels of CD85j (leukocyte immunoglobulin-like receptor B1, LILRB1), an inhibitory immune checkpoint for B cell function, were significantly lower in MM patients [34]. Reduced CD85j levels correlate with phenotypically aberrant PCs in MM patients. Decreased *CD85j* expression was detected in MGUS patients to suggest that *CD85j* loss is an early event in immune escape. Gene expression profiling of *CD85j*-overexpressing MM cells revealed a set of downregulated genes with crucial functions in MM pathogenesis and that *CD85j* overexpression increased susceptibility to T cell- and NK cell-mediated killing. Downregulation of inhibitory immune checkpoints on MM cells provides a mechanism of immune escape associated with myeloma pathogenesis [34].

### Alterations in antigen processing and presentation

**Fig. 1:**
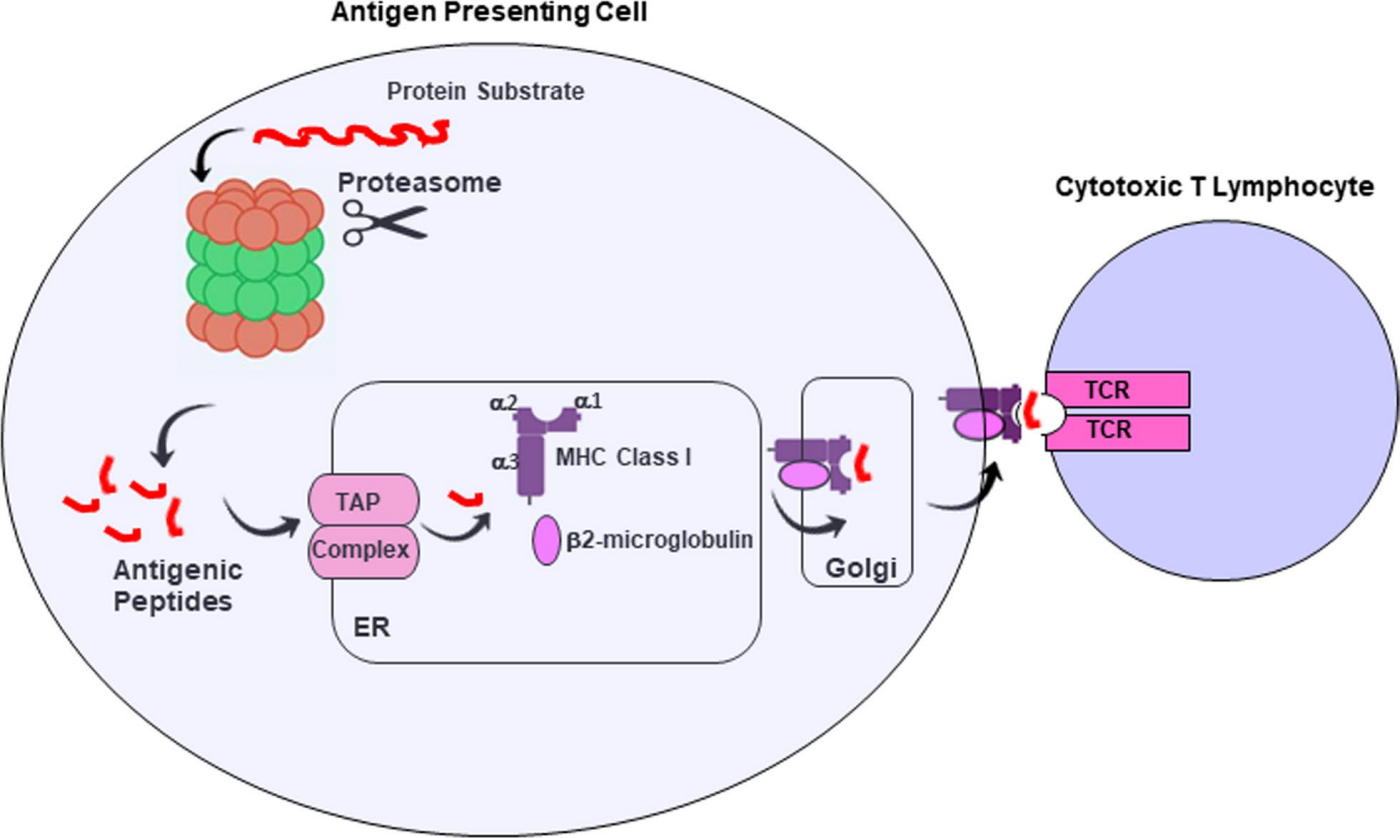
Proteasomal processing and presentation of MHC class I antigens. Proteasomes are essential for immune surveillance and cleave intracellular antigens to provide peptides that are presented on the tumor cell surface to CTLs. Proteasome are key effectors in the cascade of proteolytic processing events required for the generation of antigenic peptides. Resistance to CTLs is mediated by the loss of MHC class I expression or IFN-γ signaling within tumor cells

Immune evasion is a cancer hallmark [35, 36], and MM cells employ multiple strategies to downregulate MHC class I expression to impair CTL recognition of tumor cells (Fig. 1). Impaired antigen presentation is a highly studied mechanism of immune evasion exploited by cancer cells. Defects in the function of any of these components affects peptide production, antigen presentation and their recognition by CD8 + T cell receptors (TCRs). MM patients exhibit disruption in the presentation of class I antigens and altered expression of key players in antigen processing alters the repertoire of peptide antigens [35]. Racanelli et al. showed that malignant PCs from MM patient BM express reduced levels of antigen processing machinery (APM), i.e., constitutive and immunoproteasome subunits and TAP1/TAP2 transporters while protein chaperones, HLA class I and beta-2 microglobulin are increased in MM. Changes in the APM are associated with modified peptide repertoires to reduce the presentation of tumor-associated antigens (TAA) as well as reduced CD8 + T cell cytotoxic capacity [36].

**Fig. 2:**
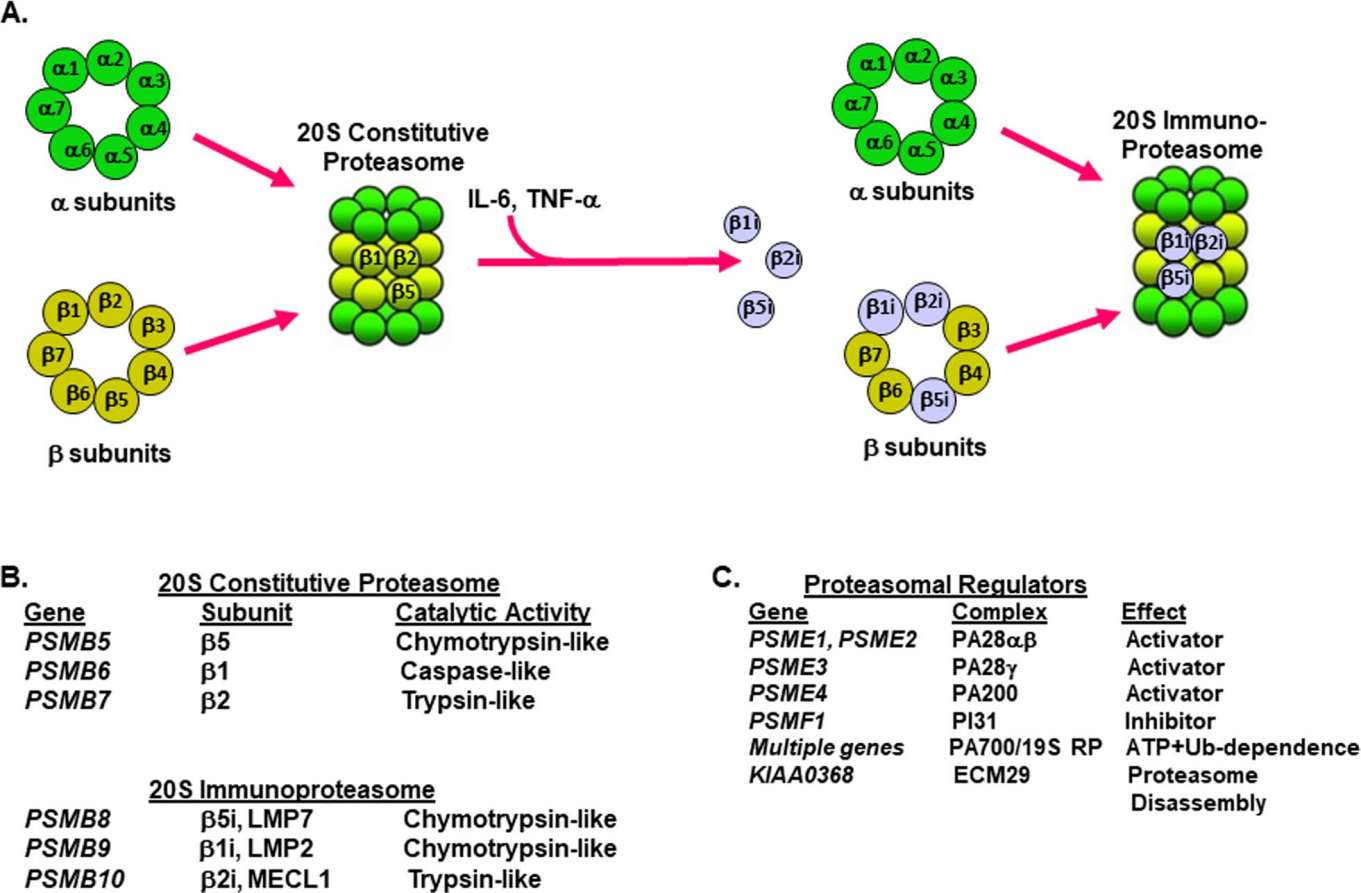
Conversion of constitutive proteasomes to immunoproteasomes. **a** Schematic representation for formation of 20S immunoproteasomes. To process antigens more efficiently, proteasomes replace some of its subunits to form immunoproteasomes. IFN-γ and TNF-α trigger transcriptional increases in IFN-γ that increase the expression of at least five immunoproteasome catalytic and activator subunits which cooperate to form 20S immunoproteasomes. New catalytic subunits (β1i, β2i, β5i) and activator subunits (PA28α/β) are incorporated into 20S constitutive proteasomes. **b** Genes that encode constitutive proteasome and immunoproteasome catalytic subunits, the catalytic activities and substrate specificities are shown. **c** Proteasome regulators that activate or inhibit proteasome-related activities are shown

Processing of MHC HLA class I antigens is accomplished through protein degradation by the constitutive proteasome as well as the immunoproteasome [37–39]. TAAs originate from the degradation of cellular proteins into short peptides that are cleaved by a specialized form of the proteasome in the cytosol known as the immunoproteasome [43]. Peptides generated by proteasomes are transported to the ER and associate with HLA class I heavy chains. IFN-γ triggers transcriptional increases in expression of at least five immunoproteasome subunits which cooperate to form immunoproteasomes (Fig. 2). New catalytic subunits (β1i, β2i, and β5i) are incorporated into 20S proteasomes to alter the catalytic specificities of proteasomes.

### Genomic alterations that reduce immunity

MM is characterized by genetic complexity and common events, e.g., hyperdiploidy, translocation of immunoglobulin heavy chain, and 13q deletion, confer an early clonal advantage [40, 41]. Patterns of genomic evolution have been characterized and subclones differentially proliferate based upon their fitness [42]. Genomic changes in MM cells are already present in PCs from MGUS patients prior to overt malignancy and immune recognition of MGUS lesions correlates with reduced risk of progression [43].

Genomic alterations within MM tumors have been shown to correlate with reduced sensitivity to a number of recently developed cell-based therapies. Homozygous deletion in chromosome 6 resulted in loss of *BCMA* (*TNFRSF17*), indicating that this mutation may play a role in CAR T resistance [44]. Using whole genome sequencing (WGS), heterozygous loss of *BCMA* in patients not exposed to BCMA CAR T cells indicated that *TNFRSF17* deletion at baseline was a risk factor for BCMA-resistance after immunotherapy [44]. Samur et al. found biallelic mutation with deletion of one copy and loss-of-function of another copy of *BCMA* as a chemoresistance mechanism in a patient with initial response to BCMA CAR T who later relapsed and developed resistance to treatment [31]. *BCMA* loss has also been reported in patients treated with BCMA-targeting bispecific antibodies. Truger et al. identified alterations in genes that encode immune targets that potentially act as biomarkers for treatment resistance seen clinically [45].

CAR T cells targeting the BCMA have resulted in deep responses in patients with relapsed MM however most remissions are not sustained. Maity et al. reported that BCL2L1 blockade of activation-induced cell death not only enhanced the viability and proliferation of BCMA-targeting CAR T cells but also reduced their functional exhaustion. The findings provide a novel approach to optimize CAR T cells and prevent T cell exhaustion [46].

Progression of MM during treatment is driven by a complex interplay between tumors and the surrounding immune microenvironment. Coffey et al. compared the cellular and humoral immunity of MM patients treated with lenalidomide maintenance to those who lost or were unable to attain minimal residual disease (MRD) negativity [47]. Patients that did not maintain MRD negativity had hallmarks of immune dysregulation at baseline and during lenalidomide maintenance, while those who achieved and sustained MRD negativity showed gradual normalization of the immune microenvironment. Exposure to high-dose melphalan (HDM) ASCT translated into cellular and humoral immunosuppression, which correlated with dynamics of MM recurrence. Independent of the impact of HDM-ASCT on host immunity, composition of the immune microenvironment varied according to the depth of response.

### Immune checkpoint inhibitors

Myeloma cells escape immunosurveillance through aberrant expression of cell surface antigens, quite different from those presented by healthy PCs [48]. MM patient tumor cells display increased expression of the immune-checkpoint receptor programmed cell death receptor ligand (PD-L1) relative to PCs from MGUS or healthy patients (Table 1). Increased PD-L1 expression is associated with reduced CTL-mediated tumor lysis, while elevated PD-L1 levels enhance regulatory T cells (Tregs) and further promote immune escape [49]. MM cells express greater levels of the inducible T cell co-stimulator ligand (ICOSL) and CD86, both of which increase T cell production of inhibitory interleukin (IL)-10 [50]. Although MM cells express CD138, there is an enriched fraction of clonogenic CD138- cells that express high levels of the embryonic marker SOX2 [51]. T cells that recognize SOX2 are lacking in MM, but are detected in MGUS patients. Increased CD28 expression has a pro-survival effect through interaction with CD80/CD86 co-stimulatory molecules and increased IL-6 production.

**Table 1:** Quantitative and qualitative changes in individual immune cell types and immune cell markers detected within the BM microenvironment of MGUS, SMM, NDMM and RRMM patients compared to BM samples obtained from healthy volunteers [113, 137, 143, 184]

Immune cells and markers	MGUS	SMM	NDMM	RRMM
CD4^+ve^ T cells	No change	No change	Reduced	Reduced
CD8^+ve^ T cells	No change	No change	No change	No change
CD4^+ve^ CD25^+ve^ Tregs	No change	No change	Increased	Increased
PD1 expression on Tregs	No change	No change	Increased	Increased
LAG3 expression on Tregs	No change	No change	No change	Increased
Granulocytic MDSC	No change	No change	Increased	Further increased
PD-L1 expression on Granulocytic MDSC	No change	No change	Increased	Increased
Monocytic MDSC	No change	No change	No change	No change
PD-L1 expression on CD138^+ve^ MM cells	No change	No change	Increased	Increased
NK cells	Slight Increase	ND	Increased	ND
NKT cells	Maintain their capacity for activation and antibody-dependent cellular toxicity	ND	Marked functional deficits	ND
Non classic CD16^+ve^ monocytes	Increased	ND	Increased	ND
Classic CD14^+ve^ monocytes	Normal/ Decreased	Decreased	Decreased	ND
Plasmacytoid DC	Decreased	ND	Decreased	ND
Monocytic DC	No change	ND	No change	ND
M2macrophages	ND	ND	Increased	Further increased
STAT3 activation in TAMS	Increased	ND	Increased	ND

No change indicates that no significant change has been reported in the literature. ND indicates that significant changes have not been determined or been reported

### Secretion of immunomodulatory molecules

MM cells express the MHC class I chain-related polypeptides A (MICA) and MICB (MICB) that function as ligands for the activating NK group 2D (NKG2D) receptor present on NK and T cells [52]. Soluble MICA is shed from MM cells and downregulates NKG2D on NK cells. Since soluble NKG2D ligands are associated with poor clinical prognosis, harnessing the NKG2D pathway has emerged as a viable strategy.

### Exosomes

Exosomes are a subset of extracellular vesicles (EVs) released by cells and have been shown to regulate the immune system. EVs are heterogeneous, present in vast numbers in the TME and exhibit immunosuppressive activity [53]. Tumor-associated exosomes facilitate tumor growth by affecting immune activation, antigen expression, and immune surveillance [54, 55]. Exosomes within the TME can directly suppress T cell activation and drive differentiation of monocytes towards myeloid-derived suppressor cells (MDSCs) that then stimulate Tregs and suppress T cell activation [56]. Tumor-derived exosomes also promote M2-like macrophage polarization to enhance tumor progression [57]. Umezu et al*.* demonstrated that miRNA-135b from MM-derived exosomes accelerated hypoxia-inducible factor (HIF)-1 transcriptional activity [58]. Exosomes upregulate nitric oxide synthase in MDSCs to enhance immunosuppression [59].

### TGF-β signaling

Transforming growth factor-β (TGF-β) plays an essential role in establishing immunological tolerance and recent studies have revealed the pro-inflammatory roles of TGF-β in inflammatory responses [60]. TGF-β also supports MM progression, the emergence of drug resistance and the progression of osteolytic bone disease [61]. TGF-β induces Foxp3-positive regulatory T cells (iTregs) as well as pathogenic IL-17-producing Th17 cells [62–64]. Vactosertib (TEW-7197) is a TGF-β type I receptor (TGF-βRI) kinase inhibitor used in combination with pomalidomide (Pom) (NCT03143985) [64, 65]. Vactosertib suppresses myeloma viability, impairs bone resorption and modulates the TME in immunocompetent mice. Efficacy assessment (PFS-6, 80%) was greater with vactosertib and Pom (PFS-6, 20%) than with Pom alone (PFS-6, 20%) or Pom with corticosteroids (PFS-6: 40%) [66].

TAAs released upon cancer cell death are processed and presented by dendritic cells (DCs) in order to prime and activate T effector cells, especially CTLs [67]. Activated tumor-specific CTLs then migrate and infiltrate the tumor bed to recognize TAAs bound on MHC class I molecules, leading to T cell-mediated cytotoxicity [68, 69]. While the presence of tumor-infiltrating lymphocytes (TILs) is associated with improved prognosis, TILs may become inactive in response to tumor-derived signals within the TME. Mutation of *β*2m, a component of the MHC-I molecule, may lead to an absence of MHC-I expression [70]. Downregulation of proteasome components and MHC complexes involved in TAA presentation may limit anti-tumor immunity.

## Tumor extrinsic mechanisms of immune escape in MM

B cell dysfunction in MM is characterized by immunoparesis, hypogammaglobulinemia and increased susceptibility to infection [71]. Effectors of anti-tumor immunity helped by antigen-presenting DCs mediate protective immune responses against tumors in healthy BM. However, in MM, tumor cells create an immunosuppressive TME which increases the number of tumor suppressive cells, e.g., Tregs, MDSCs, and reduce CTLs resulting in decreased humoral and cytotoxic immunity [71].

### T cells

Efficiently activated immune competent T cells in TMEs are required for the successful destruction of the tumor cells by the immune system. Deficiencies in T cell activity and tissue distribution have been reported in MM [72–74], while quantitative and functional impairment of T cell functioning has been described in MM and MGUS patients. Severe defects in T cell diversity has also been shown in MM patients [74, 75]. T cell subsets in MM patients are frequently abnormal and a significant reduction in the CD4/CD8 ratio has been reported in MM patients relative to healthy controls [76, 77]. Reduction in CD4 + T cells correlates with reduced progression free survival (PFS) and overall survival (OS) [78]. While the percentage of CD8+ effector cells is increased within the tumor site, these cells are functionally exhausted due to prolonged antigen exposure and are characterized by increased expression of inhibitory receptors [79]. Studies have also shown that T cells may exhibit a senescent phenotype in addition to exhaustion [80]. Soluble factors within the TME also contribute to defective T cell activity. T cells from MM patient peripheral blood (PB) demonstrate reduced IFN-γ production and tumor recognition, and defective antitumor activity in vivo. Reduced IFN-γ levels and increased IL-4 levels in patient serum indicate a shift towards Th2 polarization. IL-6 produced in the myeloma TME reduces the Th1 response [71, 81], while an increased Th1/Th2 ratio and high IFN-γ-producing T cells have been reported in the PB of MM patients [82, 83].

The CD4 + CD25 + Treg population is increased in MM patient PB and overexpression of the transcription factor *Foxp3* in CD4 + CD25 + cells correlates with immunosuppressive activity [84, 85]. Tregs from MM patients are efficient in suppressing antigen-presenting cells (APCs) as well as T cell proliferation [85, 86], while MM patients with increased Treg frequencies correlate with shorter time to progression and reduced survival [87]. Tregs from MM patients express increased amounts of TGF-β and IL-10 compared to healthy controls indicating a more suppressive phenotype [85]. Tregs also secrete granzyme B and have the capacity to kill CTLs, B cells and NK cells. Tregs suppress DC function by upregulating indolamine dioxygenease (IDO) and through interaction of CTLA-4 and Lymphocyte Activation Genes (LAG)-3 ligands present on DCs [88]. Even though IL-6 in MM increases Treg frequencies, the mechanism by which MM cells induce Tregs is poorly understood [86, 89]. Tregs decrease the clinical response upon treatment with talquetamab, a bispecific mAb (BCMA) directed against the G protein-coupled receptor GPRCD5D. Tregs reduced MM cell lysis in response to talquetamab by decreasing T cell activity and by decreased production of the pro-inflammatory cytokines IFN-γ, TNF-α and IL-2 [90].

Inflammatory Th17 cells secrete IL-17 and IL-22 and differentiate in the presence of IL-6, IL-1, IL-21 and IL-23 to express the transcription factor RAR-related orphan receptor γT (RORγt) [91]. IL-17 promotes myeloma growth and colony formation through the IL-17 receptor as well as adhesion to BM stromal cell (BMSC). Th17 cells have a reciprocal relationship with Tregs during development that is dependent upon IL-6 and TGF-β levels [92].

T cells that express CD86 and HLA-G are increased in the PB of MM patients relative to healthy controls [93]. T cells acquire tumor-derived neoantigens during trogocytosis, a unidirectional TCR-and HLA-independent process that results in immunophenotypically novel Tregs [94–96]. HLA-G + and CD86 + T cells have been shown to be immunosuppressive [97] and trogocytosis has been shown to adversely affect checkpoint inhibitor treatment [98].

### NK cells

NK cells kill MM cells by releasing lytic granules containing granzymes and perforin [99, 100]. NK cells recognize myeloma cells that downregulate the MHC I receptor and express high levels of MICA [52, 101, 102]. NK cells also reduce MM proliferation in vitro through IFN-γ and IFN-γ-deficient mice demonstrate reduced survival when injected with MM cells [2, 103].

PB from MGUS and MM patients demonstrate increased numbers of NK cells, while in advanced MM patients, the number of NK cells in PB is reduced compared to MGUS patients [104, 105]. NK cells in the TME exhibit an exhausted phenotype characterized by downregulation of NK cell activation receptors and upregulation of the programmed cell death receptor-1 (PD-1) [102, 106, 107]. NK2GD, natural cytotoxicity receptors (NCRs), and DNA accessory molecule-1 (DNAM-1, CD226) are also reduced in BM, while activation receptor 2B4 is reduced in both PB and BM [52, 108, 109]. Reduced expression of activation receptors reduce NK cell cytotoxic capabilities and reduce PC killing in spite of reduced HLA class I expression [110, 111]. Ligands expressed by PCs that help recognition by NK cells are also reduced in MM. Expression of the stress-induced ligand MICA that binds the NK cell receptor is reduced during the MGUS transition to MM. In addition, expression of ligands recognized by the NKP30 receptor are also reduced preventing the recognition and killing of myeloma cells [112]. Even at early stages of myeloma, HLA class I expression is reduced and higher levels of HLA class I expression are noted in the pleural effusions of patients with advanced MM [52, 101, 113]. HLA-E is a non-classical MHC class I molecule which plays a critical role in the immune response by both inhibiting and activating the function of NK cells through interaction with NKG2A receptors [114]. HLA-E expression correlates with worse PFS in newly diagnosed, treatment-naïve MM patients.

### B regulatory cells

Immunosuppressive B regulatory cells (Bregs) maintain immune tolerance through IL-35, TGF-β and IL-10 secretion as well as expression of inhibitory molecules, e.g., PD-L1 [115–118]. Bregs are highly heterogenous and range from immature CD24hi CD38hi cells to highly differentiated CD38 + CD27hi PCs [119]. Through IL-10 production, Bregs suppress Th1, Th2, and Th17 production, differentiation and secretion of pro-inflammatory cytokines. Bregs also induce differentiation of Foxp3 + Tregs [82, 120, 121] and inhibit antigen presentation by mononuclear cells, macrophages, and DCs [122]. Bregs are capable of suppressing anti-myeloma cell antibody-dependent cellular cytotoxicity (ADCC) by NK cells [123]. The percentage of immunosuppressive IL-10-producing Bregs is significantly increased in MM patients compared to healthy controls. Daratumumab has also been shown to reduce the number in Bregs in BM [124].

### Dendritic cells

Myeloma cells affect the generation and differentiation of DCs, and therefore, antigen presentation through direct interaction with DCs. Cytokine-mediated activation of the p38/MAPK pathway affects DC generation and differentiation in BM [125–127]. Myeloid-derived DCs in myeloma patient BM interact directly with tumor cells, downregulate proteasome subunits, and confer resistance to CD8 + T cells [128]. DCs in the TME also increase *Foxp3* expression in CD4 + T cells and increase proliferation of *Foxp3*-expressing immunosuppressive Treg cells [129].

### Tumor-associated macrophages (TAMs)

Macrophages are abundant within the TME and tumor-associated macrophages (TAMs) play a major role in MM survival and proliferation [130, 131]. MM-associated macrophages are derived from the maturation of circulating monocytes that have been recruited to the tumor [132]. TAMs are a heterogeneous population classified into M1 (classical macrophages) with immunostimulatory properties and M2 (activated macrophages) with immunosuppressive properties [133]. MM-associated macrophages display more M2-like properties with limited cytotoxicity, reduced antigen presentation, increased angiogenesis and T cell suppression [134]. Extracellular vesicles (EV) released by MM cells polarize recruited monocytes to an M2 phenotype [135]. Macrophages activate signaling pathways that aid in disease progression and the development of drug resistance by direct interaction with myeloma cells, cytokines and growth factors [134, 136]. Macrophages also directly interact with MM cells to reduce the activation and cleavage of caspase-dependent apoptosis and promote chemo-resistance [137, 138]. Myeloma-associated macrophages also are a major source of IL-6, IL-10 and IL-1β that help in tumor proliferation and survival [139, 140]. Macrophages are a source of pro-angiogenic VEGF, FGF-2 and IL-8 that promote neovascularization [134]. Macrophages from the TME exhibit a vascular, endothelial phenotype when treated with VEGF and FGF [141].

### Myeloid-derived suppressor cells (MDSCs)

MDSCs are a heterogeneous population of immature myeloid cells that accumulate in the TME as tumors progress [142–144]. MDSCs promote tumor development through immunosuppression of innate and adaptive immunity through secretion of cytokines and growth factors [145, 146]. MDSC are defined as granulocytic (G-MDSC, CD11b+, CD33+, HLADR-/low, CD14-, Lox1+) or monocytic (Mo-MDSC, CD11b+, CD33+, HLADR-/low, CD14+) [147, 148]. G-MDSCs and Mo-MDSCs are increased in MM patient PB and BM compared to healthy controls [144, 149, 150] and MDSC accumulation in PB correlates with disease activity, treatment response and relapse [143, 151].

MDSCs express immunosuppressive factors arginase, nitric oxide (NO) and reactive oxygen species (ROS) which enhance CD3 ligation that suppresses T cell proliferation and activation [152]. NO and superoxides produced by MDSCs increase nitration of TCRs in the TME to alter their binding to MHC molecules and impair antigen-specific T cell responses. MDSCs also downregulate CD62L in T cells and impair their migration to tumor sites [153]. MDSCs induce Th17 differentiation which promotes chronic inflammation and angiogenesis. IL-17 production and chronic inflammation promote recruitment of MDSC to the tumor site [154]. IL-10 produced by MDSCs induce Tregs and enhance immunosuppression at the tumor site [151]. MDSCs also promote immune escape through overexpression of PD ligands and suppress NK cell activity [155, 156]. Crosstalk between MDSC and tumor-associated macrophages promotes cytokine release to promote M2 macrophage differentiation and tumor survival [157, 158].

### High density neutrophils

Although neutrophils are the first line of host defense, recent studies have demonstrated an immunosuppressive role for high density neutrophils (HDNs) in MM [159]. Clinically, the ratio between the absolute neutrophil count (ANC) and lymphocytes has been shown to be predictive of OS and disease outcome in hematologic malignancies [160]. Neutrophils from MM patients display a different morphology, phenotype and gene expression patterns compared to those from healthy donors. HDNs express elevated levels of arginase that contribute to immunosuppression, reduced phagocytosis and less oxidative burst that may be corrected by arginase inhibitors [161]. Increased arginase expression also correlates with STAT-1 and STAT-3 signaling, to suggest an association with triggering of type 2 cytokine receptors resulting in chronically-activated neutrophils. Neutrophils in the TME show a progressive increase in autophagy and JAK/STAT signaling to support pro-inflammatory, survival signals [161, 162].

HDNs suppress T cell proliferation and activation and contribute to immunosuppression [163].

## Tumor microenvironment suppression of immune responses

**Fig. 3:**
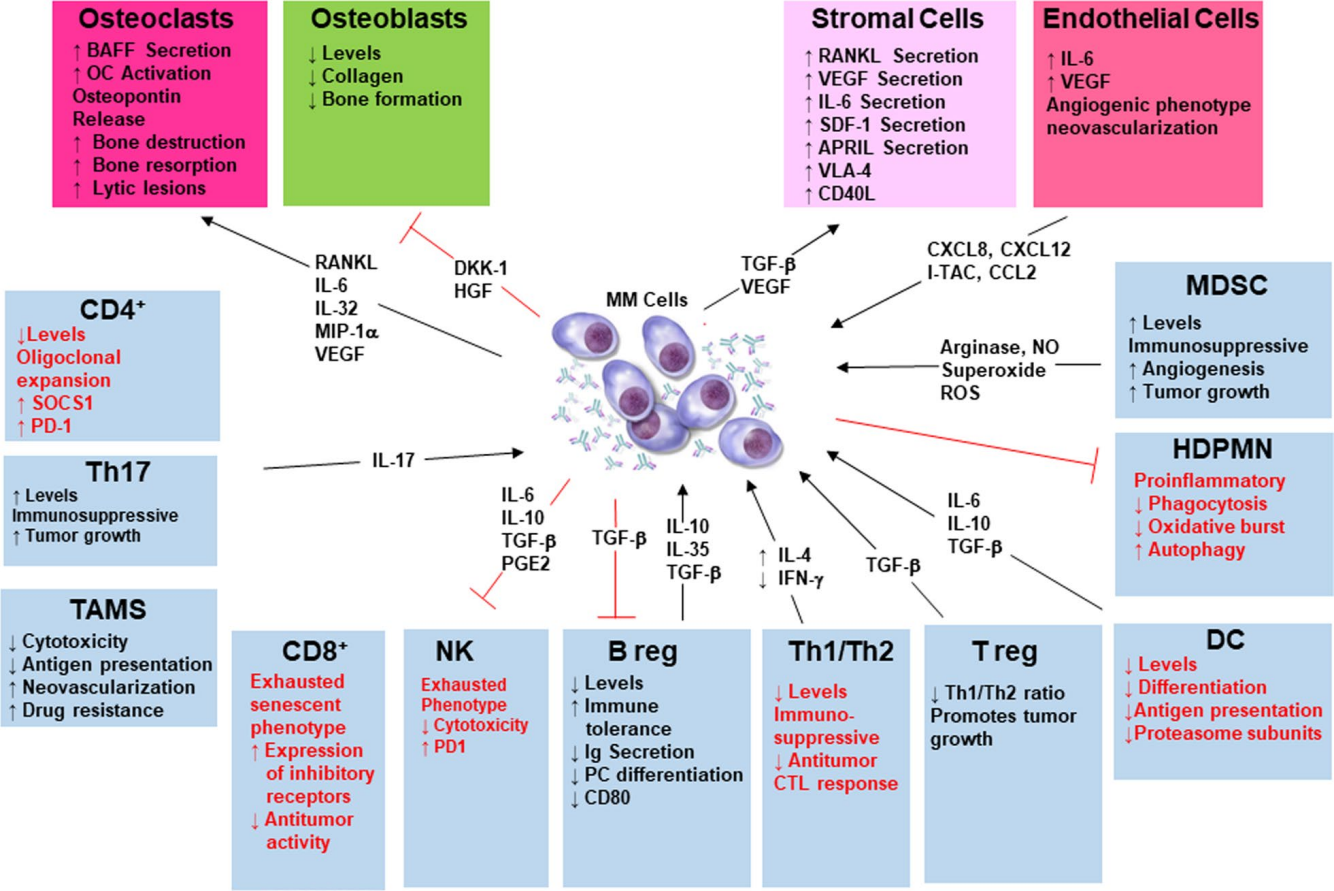
The immunosuppressive tumor microenvironment in MM. The TME present in BM creates a unique milieu that favors MM immune evasion and promotes disease progression. The tumor-immune niche and the tumor-microenvironment is implicated in malignant cell protection against anti-tumor therapy. The BM niche, composed of a cellular compartments, e.g., stromal cells, osteoblasts, osteoclasts, endothelial cells, and immune cells, an acellular compartment, e.g., extracellular matrix and liquid milieu, e.g., cytokines, growth factors, and chemokines, promote the homing differentiation, migration, proliferation, survival, and drug resistance of malignant PCs. MM cells inhibit the development of an effective anti-tumor immune responses through defects in T cell function, ineffective antigen presentation, reduced phagocytic capacity, natural killer and dendritic cell dysfunction; decreased responsiveness to IL-2 and defects in B cell immunity; upregulation of inhibitory pathways; and production of excessive proinflammatory cytokines. Immune cells including plasmacytoid dendritic cells and macrophages further trigger tumor cell proliferation, survival, and drug resistance. Novel therapies in MM target not only the tumor cell but also the BM and TME

The TME is a highly complex, continuously evolving entity that supports bidirectional, mutually beneficial communication between malignant PCs and the BM milieu (Fig. 3). The TME serves as a protective niche to promote tumor growth, drug resistance and impairs immune surveillance [164]. Hallmark features of the TME include immune and stromal cells, blood vessels, and extracellular matrix. The TME is not just a silent bystander, but rather an active promoter of cancer progression [165]. In MM, complex crosstalk between hematopoietic stem cells, myeloid cells, T and B lymphocytes, NK cells, erythrocytes, osteoclasts as well as (non-hematopoietic) osteoblasts, stromal cells, e.g., fibroblasts, endothelial cells, and acellular components, e.g., extracellular matrix and cytokines, growth factors and chemokines produced by cellular components play an integral role in tumor progression and immune resistance [12, 166].

In MM patients, the TME exhibits increased levels of IL-6, IL-10, and TGF-β that downregulate NK cell receptors and ligands. These changes contribute to functionally defective NK cells that exhibit a CD95- MHC class 1hi, MICA low phenotype [167]. IL-10 also inhibits production of the pro-inflammatory cytokines, e.g., TNF-α and IFN-γ and reduced IFN-γ levels further contribute to NK cell dysfunction. Soluble factors produced in the TME, e.g., prostaglandin E2 and indolamine hydrogenase, also inhibited NK cytotoxic activity. Hypoxia present in the BM TME further contributes to reduced NK cell responses in myeloma [168, 169].

The TME of MM can critically impair therapy outcome, including immunotherapies. Matrix proteins and BMSCs interact with MM cells to reduce immunosurveillance [170]. NF-κB-dependent adhesion of myeloma cells to BMSCs triggers IL-6 secretion to inhibit NK cytotoxicity. MM cells secrete growth factors, e.g., TGF-β and VEGF, that inhibit T, NK and DCs, promote angiogenesis and upregulate IL-6 secretion. Vascular endothelial cells along with stromal cells suppress CTL and NK cell-mediated tumor killing by deregulating components of the apoptotic machinery. BM mesenchymal stromal cells (BMMSCs) protect MM cells against the lytic activity of MM-reactive CTLs and daratumumab-redirected NK cells through upregulation of the anti-apoptotic proteins Survivin and Mcl-1. Holthoff et al. demonstrated the negative impact of the TME against immunotherapies and suggest that outcome of CAR T cell or conventional CTL therapies could benefit from inhibition of anti-apoptotic proteins upregulated in MM cells through BMMSC interactions. BMMSC-mediated protection of MM cells is not through reduction of granzyme B or IFN-γ but through upregulation of anti-apoptotic machinery and can be completely overcome by the small molecule anti-apoptotic inhibitor FL118 [171].

BM osteoclasts secrete a proliferation-inducing ligand (APRIL) to upregulate *TGF-β, IL-10* and *PD-L1* levels and enhance immunosuppression [164, 172]. APRIL binds TACI, a member of the tumor necrosis factor (TNF) superfamily expressed on Tregs, BMSCs and PCs to promote Treg viability. APRIL also increases Treg induction by MM cells to enhance Treg-mediated inhibition of T cell activity [173]. BMSC-derived exosomes further promote MM proliferation, migration, and survival, induce drug resistance and influence signaling pathways [174].

Blinatumomab can induce a complete hematological remission in patients in 46.6% with relapsed/refractory B-precursor acute lymphoblastic leukemia (r/r ALL) resulting in a survival benefit compared to chemotherapy. Only BM blast counts before therapy have shown a weak prediction of response. The frequency of Tregs, measured by CD4/CD25/FOXP3 expression, predicts the outcome of immunotherapy with the CD19-directed BITE blinatumomab [175]. Blinatumomab responders average 4.82% Tregs (CI: 1.79–8.34%) in PB, whereas non-responders demonstrated 10.25% Tregs (CI: 3.36–65.9%). Enumeration of Treg identifies r/r ALL patients with a high response rate to blinatumomab. Therapeutic removal of Tregs may convert blinatumomab non-responders to responders [175].

The efficacy of daratumumab depends partially on CD38 expression on MM cells and all-*trans* retinoic acid (ATRA) upregulates CD38 expression to revert daratumumab-resistance ex vivo. A phase 1/2 study (NCT02751255) evaluated the efficacy of daratumumab combined with ATRA in daratumumab-refractory MM. Patients that previously achieved at least a partial response or minimal response/stable disease with prior daratumumab monotherapy had a significantly longer PFS compared with patients who immediately progressed on daratumumab as single agent (median PFS 3.4 and 2.8 vs. 1.3 months). Addition of ATRA and re-intensification of daratumumab had limited activity in patients with daratumumab-refractory MM, which may be explained by transient upregulation of CD38 [176].

**Fig. 4:**
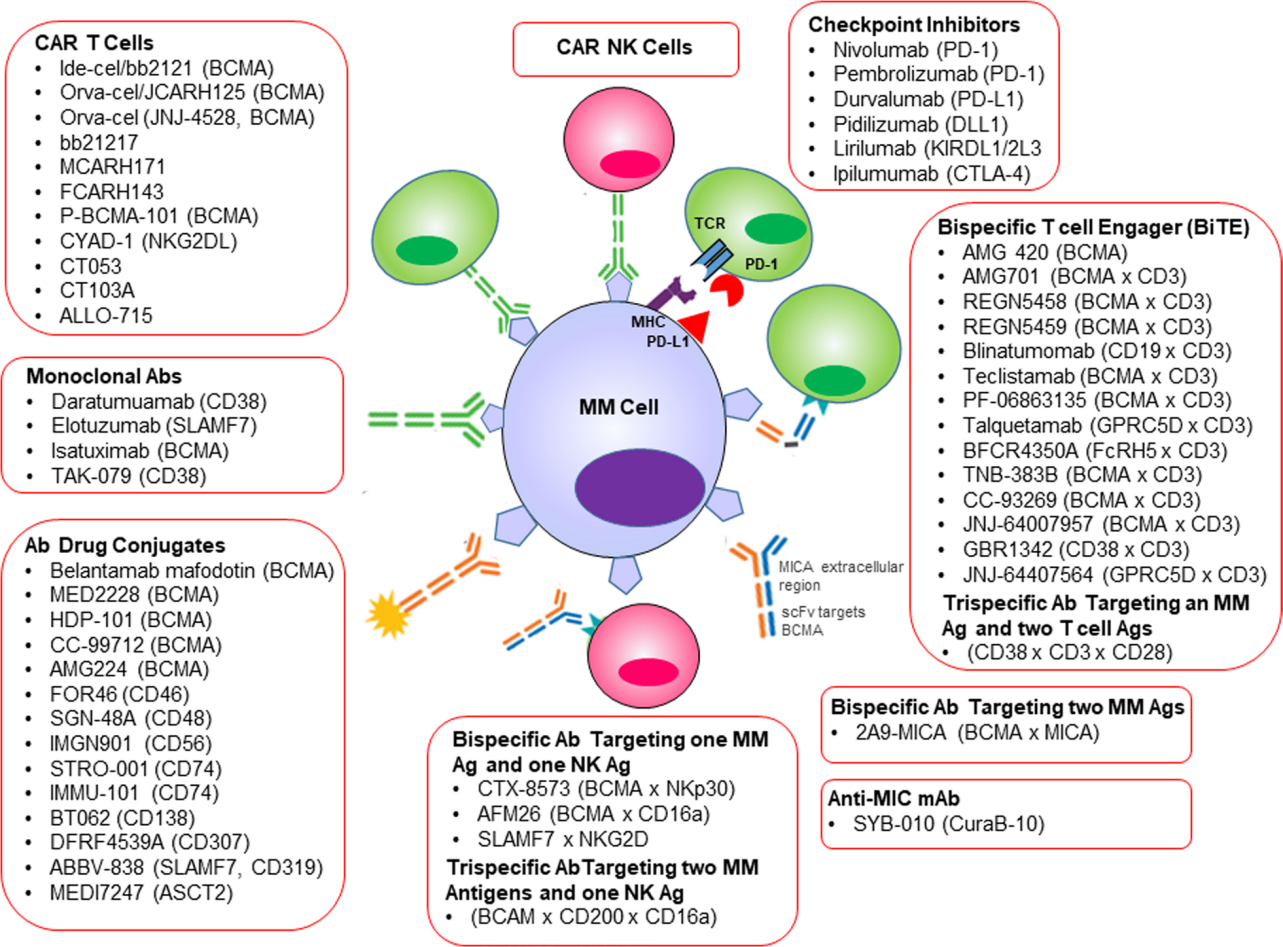
Current and emerging immunotherapeutic strategies in MM. The past two decades has seen an increase in MM patient survival. The first Ab-based FDA-approved immunotherapies, daratumumab and elotuzumab are shown. More recently FDA-approved therapies include efficacious and transformative drugs that harness the immune system. This success has been heralded by idecabtagene vicleucel, the first CAR T cell-based therapy approved for MM. Major areas of development include Ab-drug conjugates, enhancement of T cell and NK-mediated cytotoxicity through CAR T cells, BiTEs and checkpoint blockade [11–15, 178, 179, 181, 185–195]

Cell surface expression of the orphan G protein-coupled receptor, GPRC5D, is significantly greater on MM cells, compared with normal PCs, which renders it a promising target for immunotherapy. The bispecific Ab, talquetamab, effectively targets and kills GPRC5D + MM cells in the presence of T cells from healthy donors as well as heavily pre-treated patients (Fig. 4). Direct contact with BMSCs impaired the efficacy of talquetamab, while combination with daratumumab or Pom enhanced talquetamab-mediated lysis of primary MM cells [90]. Residual immature, myeloma cells within BM increase TAMS, Tregs, memory B cells and reduce PFS and OS in MM independent of cytogenetics, disease status and transplant-eligibility. Therefore, immune reconstitution in MRD negative patients may increase disease-free survival and should also be considered as a treatment strategy [47, 177].

## Strategies to overcome immune escape in MM

CAR T, CAR NK cells, Ab drug conjugates, and checkpoint inhibitors have been tested in clinical trials or are in development to treat MM (Fig. 4). BiTE therapy is an immunotherapy that works by serially killing tumor cells by placing them in proximity to T cells. The anti-CD19 X anti-CD3 drug, blinatumomab (Blincyto) (Fig. 4). CTX-8573 is a first-in-class, common light chain-based NKp30 x BCMA bispecific Ab that targets BCMA+ plasma cells and potently recruits and activates innate cells through engagement of the NKp30, as well as the activating receptor CD16a through an intact Fc. Compared to mAbs that only engage CD16a, the NKp30 bispecific platform increases ADCC potency more than 100-fold, and maintains activity in the context of CD16a downregulation (Fig. 4).

Bispecific Abs have been engineered in > 50 different formats, including dual-affinity retargeting proteins (Fig. 4). Recombinant bispecific proteins have been engineered to simultaneously bind (at least) two distinct antigens and CARs facilitate T and NK cell mediated killing of malignant cells by redirecting autologous CTLs to cell-surface tumor antigens. Importantly, BiTE and CAR T approaches are independent of endogenous TCR specificity and also independent of MHC specificity on myeloma cells. Recent clinical studies have benefitted from more efficacious immunotherapeutic agents and treatment strategies and yield improved outcomes. Despite these advances, the efficacy of most cancer immunotherapies has been modest. A recurring scenario is that therapeutic maneuvers initially lead to measurable anti-myeloma clinical responses but ultimately failed to improve OS. Immunotherapeutics have been developed that alleviate immunosuppression, e.g., IMiDs and immune checkpoint inhibitors, target highly selective antigens in the form of mAbs that stimulate immune cells to selectively kill malignant cells, e.g., CAR T cells, BiTEs [16, 178–181].

Strategies to enhance the priming phase by loading DC *ex vivo* or *in vivo* with tumor antigens, peptide- or DNA-vaccines, or TLR agonists are in development [181, 182]. Restoring or forcing antigen presentation by MM cells themselves represents an alternative to DC-based therapies to enhance priming. Tumor cells are more numerous than DCs in BM and could directly present their own endogenous antigens, without the need for cross-presentation by DCs. Tumors evade immunosurveillance through shedding the MHC class I chain-related protein A and B (MICA/B) [29]. MICA/B function as ligands for NKG2D, an activating receptor on NK and T cells. Shedding reduces MICA/B levels on MM cells, masks the NKG2D receptor and impairs tumor recognition [102]. Tumor-derived soluble NKG2D ligands are associated with poor clinical responses to PD1/PD-L1 blockade therapy [183]. SYB-010 is a first-in-class, immune stimulatory mAb that targets tumor-released soluble MICA/B (sMIC) (Fig. 4).

In the related B cell malignancy acute lymphoblastic leukemia (ALL), primary resistance to CD19-directed CAR T cell therapy (CART19) occurs in 10–20% of patients and CART19 resistance is a significant barrier to treatment [184]. A genome-wide loss-of function screen, found that impaired death receptor signaling in ALL led to rapidly progressive disease despite CART19 treatment. The effect was mediated by an inherent resistance to T cell cytotoxicity that permitted antigen persistence and was magnified by CAR T cell impairment. Notably, the findings were validated using samples from two CAR T cell clinical trials, where the authors found that reduced expression of death receptor genes was associated with worse OS and reduced T cell fitness.

## Conclusions

MM remains an incurable malignancy despite great advances in therapy. Residual disease persists and reaches an equilibrium with host immunity that results in either sustained remission or disease recurrence. During this equilibrium, chemotherapy, immune adjuvants, and consequences of therapy-induced responses, e.g., proinflammatory cytokines, shift the balance in either direction. Novel therapies must balance immune-enhancing effects while minimizing side effects. IMiDs, PI’s, mAbs, checkpoint inhibitors, NK and T cell-based therapies have the potential to reverse immunosuppression and restore effective immunosurveillance. Since the pathology of MM includes a critical interaction between tumor cells and the TME, targeting this interaction should provide clinical benefit. Most studies thus far have been performed with RRMM, however, it is not known if patients would benefit from strategies at early disease stages. Enhanced knowledge of tumor immunity should help design strategies that improve OS.

## Data Availability

Not applicable.

## Abbreviations

ADCC: Antibody-dependent cellular cytotoxicity
ALL: Acute lymphoblastic leukemia
ANC: Absolute neutrophil count
APC: Antigen-presenting cell
APM: Antigen presenting machinery
APRIL: A proliferation-inducing ligand
ASCT: Autologous stem cell transplantation
ATRA: All-*trans* retinoic acis
β2m: β2-Microglobulin
BiTEs: Bispecific T-cell engagers
BM: Bone marrow
BMSC: Bone marrow stromal cell
BMMSC: Bone marrow mesenchymal stromal cells
B reg: Regulatory B cell
CAR: Chimeric antigen receptor
CART19: CD19-directed CAR T cell therapy
CD: Cluster of differentiation
CR: Complete remission
CTL: Cytotoxic T lymphocyte
DC: Dendritic cell
DNA: Deoxyribonucleic acid
DNAM-1: DNA accessory molecule 1
ER: Endoplasmic reticulum
EV: Extracellular vesicle
FGF-2: Basic fibroblast growth factor
GABARAP: Gamma-aminobutyric acid receptor-associated protein
G-MDSC: Granulocytic myeloid-derived suppressor cell
HDN: High density neutrophil
HIF-1: Hypoxia-inducible factor 1
HLA: Human leukocyte antigen
ICD: Immunogenic cell death
ICOSL: Inducible T cell co-stimulator ligand
IDO: Indolamine dioxygenase
IFN-γ: Interferon gamma
IL: Interleukin
IMiDs: Immunomodulatory agents, immunomodulatory drugs
iTreg: Foxp3-positive regulatory T cell
mAb: Monoclonal antibody
mRNA: Messenger ribonucleic acid
MAPK: Mitogen-activated protein kinase
MDSC: Myeloid-derived suppressor cell
MGUS: Monoclonal gammopathy of unknown significance
MHC: Major histocompatibility complex
MICA: Major histocompatibility complex class I related chain A
MICB: Major histocompatibility complex class I related chain B
MM: Multiple myeloma
MRD: Minimal residual disease
Mo-MDSC: Monocytic myeloid-derived suppressor cell
NCR: Natural cytotoxicity receptor
NCT: National clinical trial
NDMM: Newly diagnosed multiple myeloma
NF-κB: NF-kappa B
NK: Natural killer
NO: Nitric oxide
NS-SNV: Non-synonymous single nucleotide variation
OS: Overall survival
PB: Peripheral blood
PC: Plasma cell
PD-1: Programmed cell death protein 1
PD-L1: Programmed cell death ligand 1
PFS: Progression-free survival
PI: Proteasome inhibitors
PMN: Polymorphonuclear
Pom: Pomalidomide
ROR: RAR-related orphan receptor
ROS: Reactive oxygen species
RR: Relapsed and/or refractory
SEER: Surveillance, epidemiology and end results
SMM: Smoldering multiple myeloma
sMIC: Soluble MICA or MICB
TAA: Tumor-associated antigens
TAP: Transporter associated with antigen processing
TCR: T cell receptor
TGF-β: Transforming growth factor beta
TGF-βRI: Transforming growth factor beta type I receptor
TIL: Tumor-infiltrating lymphocyte
TLR: Toll-like receptor
TME: Tumor microenvironment
TNF: Tumor necrosis factor
Treg: Regulatory T cell
VEGF: Vascular endothelial growth factor
WGS: Whole genome sequencing
